# Genotyping Study of *Salmonella* 4,[5],12:i:- Monophasic Variant of Serovar Typhimurium and Characterization of the Second-Phase Flagellar Deletion by Whole Genome Sequencing

**DOI:** 10.3390/microorganisms8122049

**Published:** 2020-12-21

**Authors:** Ainhoa Arrieta-Gisasola, Aitor Atxaerandio-Landa, Victoria Garrido, María Jesús Grilló, Ilargi Martínez-Ballesteros, Lorena Laorden, Javier Garaizar, Joseba Bikandi

**Affiliations:** 1 Immunology, Microbiology and Parasitology Department, Faculty of Pharmacy, Bioaraba Health Research Institute, University of the Basque Country (UPV/EHU), 01006 Vitoria-Gasteiz, Spain; ainhoa.arrieta@ehu.eus (A.A.-G.); aitor.achaerandio@ehu.eus (A.A.-L.); ilargi.martinez@ehu.eus (I.M.-B.); javier.garaizar@ehu.eus (J.G.); joseba.bikandi@ehu.eus (J.B.); 2 Animal Health Group, Institute of Agrobiotechnology (CSIC-Navarra Government), 31192 Mutilva, Spain; victoria.garrido@csic.es (V.G.); mj.grillo@csic.es (M.J.G.)

**Keywords:** *Salmonella*, monophasic, deletion, IS26 insertion, typing, whole genome sequencing

## Abstract

After *Salmonella* Enteritidis and *S.* Typhimurium, *S.* 4,[5],12:i:- is the most reported serovar in human clinical cases. During the past 20 years, many tools have been used for its typing and second-phase flagellar deletion characterization. Currently, whole genome sequencing (WGS) and different bioinformatic programs have shown the potential to be more accurate than earlier tools. To assess this potential, we analyzed by WGS and in silico typing a selection of 42 isolates of *S*. 4,[5],12:i:- and *S*. Typhimurium with different in vitro characteristics. Comparative analysis showed that SeqSero2 does not differentiate *fljB*-positive *S*. 4,[5],12:i:- strains from those of serovar Typhimurium. Our results proved that the strains selected for this work were non-clonal *S*. 4,[5],12:i:- strains circulating in Spain. Using WGS data, we identified 13 different deletion types of the second-phase flagellar genomic region. Most of the deletions were generated by IS26 insertions, showing orientation-dependent conserved deletion ends. In addition, we detected *S*. 4,[5],12:i:- strains of the American clonal line that would give rise to the Southern European clone in Spain. Our results suggest that new *S*. 4,[5],12:i:- strains are continuously emerging from different *S*. Typhimurium strains via different genetic events, at least in swine products.

## 1. Introduction

*Salmonella enterica* subs. *enterica* consist of more than 2600 serovars [1]. Nontyphoidal *Salmonella* serovars are common causative zoonotic agents of bacterial food-borne disease worldwide. After *S*. Enteritidis and *S*. Typhimurium, the monophasic variant *S*. 4,[5],12:i:- is the most frequently reported in clinical human infections, and is responsible for about 4.7% of total reported cases [2]. The monophasic variant *S*. 4,[5],12:i:- is antigenically similar to *S.* Typhimurium (which has the antigenic formula 4,5,12:i:1,2) but does not express the second-phase flagellar antigen, which is identified as 1,2 in the *S.* Typhimurium antigenic formula. The first described monophasic variant of *S*. Typhimurium emerged in Spain in 1997 [3] and became the fourth most common serovar in clinical isolates in 1998 [4]. Thereafter, the emergence of multiple clones of monophasic variant of *S.* Typhimurium has been reported worldwide [5]. According to data from seven European countries, reported to The European Surveillance System (TESSy), the clinical isolates of *S.* 4,[5],12:i:- increased from 360 in 2007 to 1416 in 2009 [5], consequently, this serovar was included in the European *Salmonella* control program. In 2018, 2553 cases were confirmed, associated mainly with the consumption of broiler (43.4%) and pig-derived products (39.6%) [2]. Specifically in Spain, *S.* 4,[5],12:i:- is still the third most common serovar with 126 cases reported in 2016 [6].

Given the high incidence of the *S.* 4,[5],12:i:- serovar, many research groups have carried out analyses of the deletions involving the second-phase flagellar genes, suggesting that multiple independent clones may be emerging in the United States and Europe [7]. Spanish isolates belong to the first described clone, which were: (i) U302 phage type, (ii) multi-resistant to ampicillin, chloramphenicol, sulphonamide, gentamicin, streptomycin, tetracycline and sulfamethoxazole-trimethoprim (ACSuGSTSxT profile), (iii) defective in 16 to 54 genes upstream of the *iroB* gene (including the lack of *fljAB* operon) and (iv) positive in the presence of the insertion sequence IS26 [4,8,9]. By 2002 and 2003, additional phage types began to be recognized, suggesting divergence from the original clone or introduction of new *S.* 4,[5],12:i:- lineages around the world, evolved through multiple independent events, most likely from *S.* Typhimurium ancestors [4,7,8,10,11,12,13].

A second clonal line was proposed by Soyer et al. in the United States (US), in 2009 [10]. These isolates lacked 77 genes from STM2692 to STM2772 and harbored a 7 kb fragment insertion composed of two partial Fels-2 genes (STM2704 and STM2706) and three genes homologous to STM1054, STM1053 and STM1997 (*umuC*), which encode two Gifsy-2 prophage genes and a component of DNA polymerase V (*umuC*) [10]. Later, in 2014, Mourão et al. proposed the Southern Europe clone to classify Portuguese origin strains that contained the same particular genomic deletion as the US strains described by Soyer et al. but showed resistance to chloramphenicol, streptomycin, sulfamethoxazole, tetracycline and trimethoprim (CSSuTTm) caused by the acquisition of IncR plasmids [14]. The authors suggested a common evolutionary origin for the US clone and Southern European clone, with the acquisition of IncR plasmids by the latter, possibly driven by antibiotic selective pressure and availability of IncR in the European metagenome [14].

Another clonal line is composed of a R-ASSuT monophasic strains, with the deletion of the *fljB* gene and which was detected in other European countries [7,12,15,16,17]. Moreover, in the latter clone, the presence of IS26 in the same position as the *fljAB* operon in many monophasic strains has been reported, supporting the hypothesis that the insertion sequence IS26 recognizes a hotspot in the second-phase flagellar genomic region [16,18]. Furthermore, it has been seen that this area has a lower average GC content in comparison with the *Salmonella* core genome (45 vs. 52.2%), supporting the idea that it could be an integration hotspot for foreign DNA [18]. Even so, little is known about the preference of IS26 towards that hotspot located in the second-phase flagellar genomic region. In addition to these clones that have the entire *fljB* deletion, strains possessing a *fljB* coding sequence (*fljB*-positives) have also been described in several European countries in recent years [19,20,21]. The emergence of these strains has been the result of other deletions, insertions or mutations in the *fljAB* operon [21].

To date, next generation sequencing (NGS) techniques have not been used for genetic typing of the *S.* 4,[5],12:i:- strains isolated in Spain. The genetic study of the second-phase flagellar region of *S*. 4,[5],12:i:- strains will allow us to determine if the characterized monophasic strains belong to some of the clones described above, or if the emerging variations suggest multiple clonal lineages. Therefore, the aims of this study were to analyze the *Salmonella* 4,[5],12:i:- serotype by whole genome sequencing (WGS) and bioinformatic tools, to characterize the deletion of the second-phase flagellar genomic region and to establish the genetic relationship between *S*. 4,[5],12:i:- and *S*. Typhimurium strains.

Here, we used WGS data and various bioinformatic tools that allowed us to recapitulate many of the results of standard microbiological typing assays. Additionally, our findings suggest that the genetic events leading to the emergence of the *S*. 4,[5],12:i:- monophasic serotype involved several lineages and not the expansion of a single clone. This research shows how new *S*. 4,[5],12:i:- monophasic strains are emerging from *S*. Typhimurium with no close phylogenetic relationship. Furthermore, this research is a clear example of the usefulness of NGS techniques to carry out a complete characterization of *S*. 4,[5],12:i:- and it shows that these techniques can be useful for the monitoring of *S*. 4,[5],12:i:- strains circulating both in Spain and worldwide.

## 2. Materials and Methods

### 2.1. Isolate Collection

A total of 42 *Salmonella enterica* isolates collected from 1999 to 2015, from different matrixes and Spanish locations, were selected for this study (Table A1). The selection was made based on the different origins and characteristics studied in vitro with the aim of reflecting the genetic variations among the monophasic strains circulating in recent years in Spain. Briefly, the isolates were: (i) 13 *S*. 4,[5],12:i:- from unrelated gastroenteric infection cases; (ii) 4 *S*. 4,[5],12:i:- from pork sausages; (iii) 15 *S*. 4,[5],12:i:- of asymptomatic pigs, of which 13 were from the intestinal content (IC) and 2 from mesenteric lymph nodes (MLNs); and (iv) 10 *S*. Typhimurium, of which 9 were from MLNs and 1 was from the IC of asymptomatic pigs. All the isolates were provided with the serotyping determined by the Kauffmann–White scheme [1], the antimicrobial susceptibility determined by the Kirby–Bauer disc diffusion test [22] and phage type defined according to Anderson et al. [23].

### 2.2. Whole Genome Sequencing and in Silico Genotyping

Genomic DNA from the 42 isolates was extracted and purified using the NucleoSpin Tissue DNA purification kit (Macherey-Nagel, Duren, Germany), according to the manufacturer’s instructions. Sequencing libraries were prepared using the NexteraXT library preparation kit and WGS was performed on the Illumina MiSeq platform, generating 250 bp paired-end reads. The sequences were submitted to the European Nucleotide Archive (https://www.ebi.ac.uk/ena) under the project accession number PRJEB37694. Raw reads were assembled into contigs using the INNUca pipeline (https://github.com/theInnuendoProject/INNUca), which consists of several modules [24]. Firstly, INNUca calculates whether the sample raw data fulfil the expected coverage (minimum 15X). Then, INNUca uses FastQC (https://www.bioinformatics.babraham.ac.uk/projects/fastqc/) to perform a read quality analysis and Trimmomatic [25] to trim the reads. After subjecting reads to quality analysis using FastQC again, INNUca proceeds to de novo draft genome assembly with SPAdes [26]. Subsequently, coverage filtering is performed using Bowtie (http://bowtie-bio.sourceforge.net/index.shtml) and Samtools (http://www.htslib.org/doc/samtools.html). Next, Pilon [27] improves the draft genome, removing very poorly represented sequences, correcting bases, fixing misassemblies and filling gaps. Finally, the INNUca workflow ends with species confirmation and MLST prediction of seven genes using mlst2 (https://github.com/tseemann/mlst).

Serovar and antibiotic resistance prediction was performed using SeqSero2 [28] and ResFinder 4.0 (95% ID threshold, 60% minimum length) [29], available as a web service at the Center for Genomic Epidemiology (http://www.genomicepidemiology.org), and was then compared with those provided by classical microbiology.

Pathogenicity island, plasmid and prophage sequence prediction was performed using SPIFinder 1.0 (95% ID threshold) (https://cge.cbs.dtu.dk/services/SPIFinder), PlasmidFinder 2.1 (95% ID threshold) [30] and PHASTER (90% ID threshold) [31], respectively. The IS26 insertion sequence was detected by in silico PCR simulation [32].

The core genome MLST profile was analyzed using cgMLSTFinder 1.1 (https://cge.cbs.dtu.dk/services/cgMLSTFinder/) based on the Enterobase scheme [33] consisting of a core of 3002 genes.

### 2.3. Characterization of the fljAB Operon Deletions by WGS

The presence or absence and the deletion ends of the *fljAB* operon in *S.* 4,[5],12:i:- were determined on contigs by in silico PCR simulation [32]. *Salmonella* Typhimurium LT2 strain (GeneBank accession number AE006468.2) was used as a reference. Conventional PCRs were performed to corroborate the in silico characterization of the *fljAB* operon deletions. All the detected insertions were identified and annotated using BLASTn (https://blast.ncbi.nlm.nih.gov/Blast.cgi) and Clustal Omega (https://www.ebi.ac.uk/Tools/msa/clustalo/).

## 3. Results

### 3.1. Characterization of S. 4,[5],12:i:- and S. Typhimurium by WGS and Bioinformatic Tools

WGS-based genotyping results obtained by INNUca MLST, SeqSero2, ResFinder, PlasmidFinder, PHASTER, IS26 in silico PCR and cgMLSTFinder are shown in Table 1. Molecular serotyping by SeqSero2 software confirmed phenotypical serotype in 39/42 (92.86%) strains in the blind study (Table 2). The remaining 3 isolates were serotyped as *S*. Typhimurium (4,[5],12:i:1,2) by SeqSero2, while they were serotyped as *S*. 4,[5],12:i:- by agglutination serology, suggesting a lack of flagellar antigen expression in vitro in the serotyping conditions.

ResFinder allowed the detection of at least one antibiotic resistance gene in all *S*. 4,[5],12:i:- and *S*. Typhimurium strains (Table 2). The aminoglycoside family genes (i.e., *aac, aad* and/or *aph*) were the most common, with the cryptic gene *aac(6′)-laa* being found in all the isolates studied (Table 2). In addition, 14 different genotypic antimicrobial profiles were determined and 66.67% of the strains showed resistance genes to at least 3 antibiotics. The most frequent genotypic multi-resistance profile was ASSuT (19.05%). Interestingly, all the strains with this tetra-resistance profile were *S*. 4,[5],12:i:-, coinciding with the reported European monophasic clone. ResFinder correctly identified 92.12% (117/127) of the antibiotic resistances found phenotypically. Comparing with classical antibiograms, ResFinder detected the same antibiotic resistances or more in 85.71% (36/42) of the isolates. In the remaining 6 isolates, ResFinder detected fewer antibiotic resistances than in classical antibiograms.

SPIFinder found 8 different *Salmonella* pathogenicity islands (SPI-1, SPI-2, SPI-3, SPI-4, SPI-5, SPI-9, SPI-13 and SPI-14) and the Centisome 63 pathogenicity island (C63PI) (Table 1). SPI-5, which encodes the effector proteins for SPI-1 and SPI-2, was the only common pathogenicity island detected in all strains. SPI-1 and/or SPI-2 were detected together with SPI-5 in 95.24% of the strains. In two strains, neither SPI-1 nor SPI-2 were detected, even when SPI-5 was present. SPI-9, which encodes a type I secretory apparatus and a large repeats-in-toxin (RTX)-like protein, was detected in only one monophasic strain (code 749) from swine intestinal content. In addition, 73.81% of the strains showed at least 7 different pathogenicity islands.

PlasmidFinder detected plasmid replicons in most (85.71%) of the strains (Table 1). The replicon most frequently identified among monophasic strains was IncQ1 (46.88%). In contrast, among *S*. Typhimurium strains, the replicon most frequently identified was IncFII(S) (70.00%). All the genomes analyzed showed at least one prophage sequence by PHASTER analysis (Table 1). A total of 15 different prophage sequences were found, Gifsy1 (88.10%), Gifsy2 (50.00%) and Sal3 (47.62%) being the most frequent (Table 1). In silico PCR simulation detected the presence of the insertion sequence IS26 in 96.87% of the monophasic strains and in 30.00% of *S*. Typhimurium strains (Table 1).

Using a traditional *Salmonella* MLST scheme, formed by 7 housekeeping genes (*aroC, dnaN, hemD, hisD, purE, sucA* and *thrA*), the isolates were classified into 2 different sequence types (Table 1). On the one hand, most of the *S*. 4,[5],12:i:- (78.13%) were classified in ST-34 and the rest (21.87%) in ST-19. On the other hand, 90.00% of the *S*. Typhimurium strains were classified in ST-19 and the other 10.00% in ST-34. In contrast, by the cgMLST scheme consisting of 3002 genes, the isolates were classified into 23 different sequence types. None of the *S*. 4,[5],12:i:- strains shared the same cgMLST type with the *S*. Typhimurium strains, even though they were strains isolated from the same pig farm.

### 3.2. Characterization of the fljAB Operon Deletion Types by WGS

**Figure A1:**
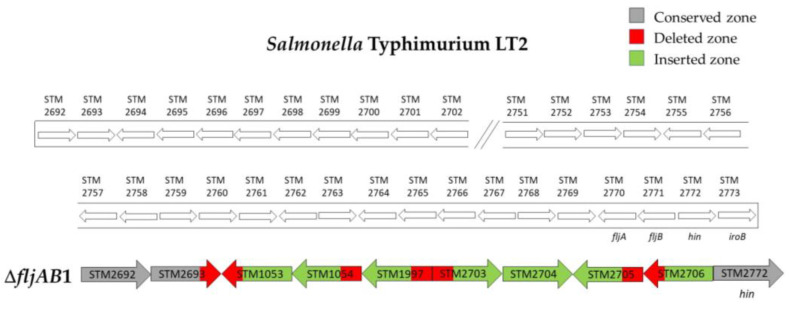
Structure of the Δ*fljAB*1 deletion type. The Δ*fljAB*1 deletion (strains 692, 697, 702 and 705) started at the STM2693 gene, where only the last two nucleotides were lacking, and ended at the intergenic region between *fljB* and *hin*, specifically 10 nucleotides downstream of the *fljB* gene. Sequencing showed an insertion of 5654 bp (colored in green) which entirely matched with the *Salmonella* Typhimurium 08-1736 strain (GeneBank accession number CP006602), containing different fragments encoding for Gyfsy-2 prophage proteins (841 bp of STM1053 and STM1054 genes), UMUC protein (540 bp of STM1997 gene) and two fragments encoding for three Fels-2 prophage proteins (777 bp from STM2703 to STM2705, and 292 bp of STM2706 gene).

**Figure A2:**
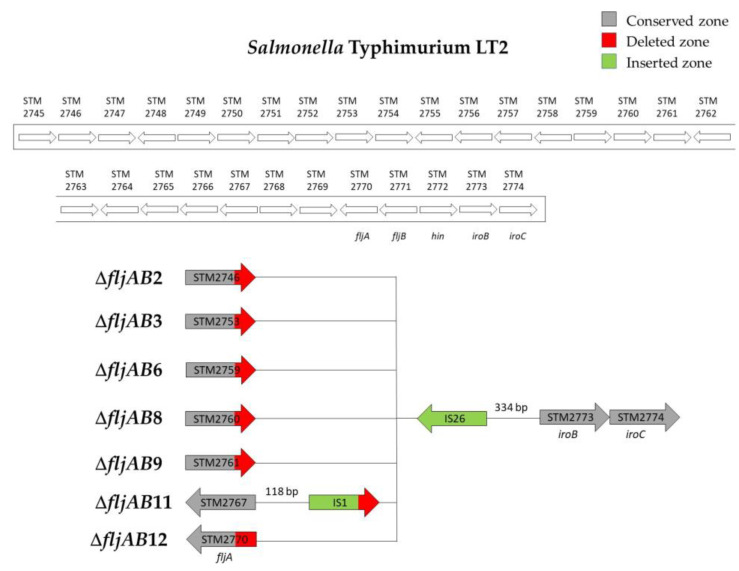
Structures of the Δ*fljAB*2, Δ*fljAB*3, Δ*fljAB*6, Δ*fljAB*8, Δ*fljAB*9, Δ*fljAB*11 and Δ*fljAB*12 deletion types. These deletion types had different starting points, but the same ending point of the deletion at the intergenic zone between *hin* and *iroB* genes (334 nucleotides upstream of *iroB*) and at least one IS26 inserted (colored in green). The Δ*fljAB*2 deletion started at the STM2746 gene. In the variant Δ*fljAB*2-A (strain 753), the deletion started at nucleotide 1201 (the last 87 nucleotides of STM2746 were deleted), and the variant Δflj*AB*2-B (strain 758) at nucleotide 1263 (the last 25 nucleotides of STM2746 were deleted). The Δ*fljAB*3 deletion started at the STM2753 gene. In the variant Δ*fljAB*3-A (strain 743), the deletion began at nucleotide 177 (the last 844 nucleotides of STM2753 were deleted), the variant Δ*fljAB*3-B (strain 746) began at nucleotide 207 (the last 814 nucleotides of STM2753 were deleted) and the variant Δ*fljAB*3-C (strain 744) began at nucleotide 353 (the last 668 nucleotides of STM2753 were deleted). The Δ*fljAB*6 deletion started at the STM2759 gene. In the variant Δ*fljAB*6-A (strain 695), the deletion started at nucleotide 1079 (the last 179 nucleotides of STM2759 were deleted) and in the variant Δ*fljAB*6-B (strains 698 and 699), the deletion started 142 bp downstream of the STM2759 gene. The Δ*fljAB*8 deletion (strains 748, 749 750, 751, 752, 754 and 757) started at nucleotide 88 of the STM2760 gene (the last 64 nucleotides were deleted). The Δ*fljAB*9 deletion (strain 755) started at nucleotide 175 of the STM2761 gene. The Δ*fljAB*11 deletion (strain 701) started at 118 bp downstream of the STM2767 gene. In the inserted fragment (colored in green), a truncated IS1 lacking 133 nucleotides and a completed IS26 were detected. Finally, the Δ*fljAB*12 deletion (strain number 747) started at nucleotide 155 of the *fljA* gene (the last 385 nucleotides were deleted).

**Figure A3:**
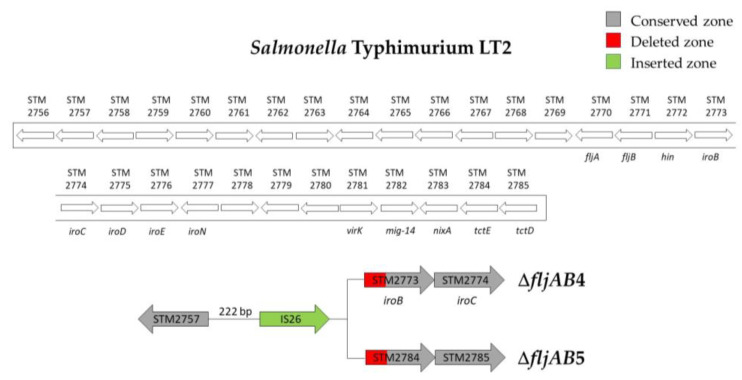
Structures of the Δ*fljAB*4 and Δ*fljAB*5 deletion types. These deletion types had the same starting point of the deletion at the intergenic zone between STM2757 and STM2758 (222 bp downstream from the STM2757) but different ending points. The Δ*fljAB*4 deletion (strains 712, 713, 714) ended at nucleotide 571 of the *iroB* gene. The Δ*fljAB*5 deletion (strain 711) ended at nucleotide 848 of the STM2784 gene. The insertion sequence IS26 (colored in green) was detected in the middle of the deletions.

**Figure A4:**
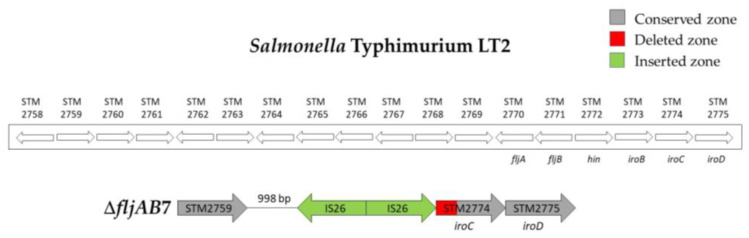
Structure of the Δ*fljAB*7 deletion type. The Δ*fljAB*7 (strain 745) deletion started 998 bp downstream of the STM2759 gene and ended at nucleotide 475 of the *iroC* gene. Two IS26 insertion sequences in opposite directions (colored in green) were detected in the middle of the deletion.

**Figure A5:**
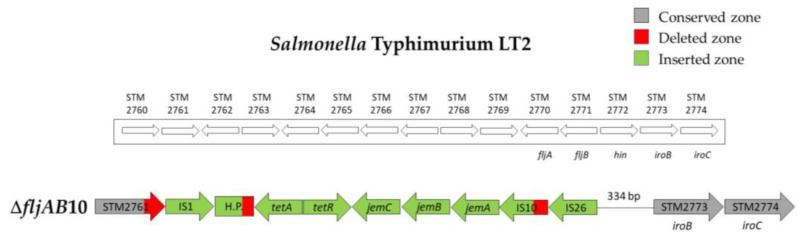
Structure of the Δ*fljAB*10 deletion type. The Δ*fljAB*10 deletion (strains 703 and 704) started at nucleotide 1125 of the STM2761 gene and ended at the intergenic zone between *hin* and *iroB* genes (334 nucleotides upstream of *iroB*). The inserted fragment (colored in green) started with an IS1, 354 nucleotides from the protein COG1309 described in the *S*. Typhimurium T000240 strain, and after that the *tetA*, *tetR*, *jemC*, *jemB* and *jemA* genes appear and a truncated IS10 lacking 973 nucleotides by an IS26.

**Figure A6:**
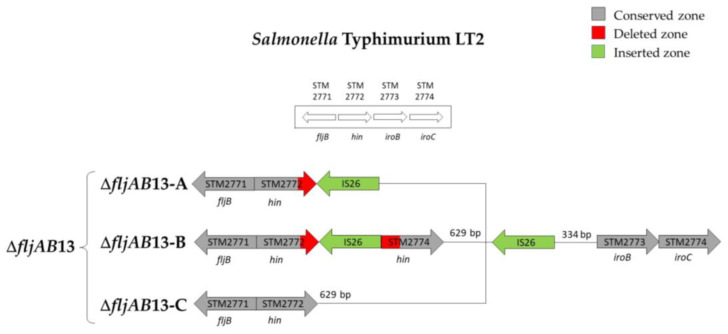
Structures of the Δ*fljAB*13 deletion type. The Δ*fljAB*13 deletion had the entire *fljB* gene present. In all variants, at least one IS26 (colored in green) was detected between *hin* and *iroB,* 334 nucleotides upstream of the *iroB* gene. That IS26 had a flanking 8 bp duplication (ATCAATAC). In the variant Δ*fljAB*13-A (strain 696), the last 33 bp of *hin* were deleted by a second IS26. In the variant Δ*fljAB*13-B (strain 694), *hin* was interrupted by a second IS26 with a flanking 8 bp duplication (GTCGAGCG). In the variant Δ*fljAB*13-C (strain 693), all genes belonging to the operon *fljAB* (*fljA*, *fljB* and *hin*) were complete. The only inserted IS26 was the one that was shared with the other two variants.

As mentioned above, the *fljAB* operon (*fljA*, *fljB* and *hin* genes) and the flanking genes from the *S*. Typhimurium LT2 genome were used as a reference to characterize the deletions. The area searched began at STM2693 and ended at STM2774. Thirteen different deletion types were characterized (Table 3 and Figure A1, Figure A2, Figure A3, Figure A4, Figure A5 and Figure A6). Twelve *fljB*-negative deletion types (*ΔfljAB*1-12) were sorted according to the deletion length (*ΔfljAB*1 being the longest and *ΔfljAB*12 the shortest). One *fljB*-positive deletion type was detected with different variations included (*ΔfljAB*13).

The *ΔfljAB*1 type was the longest deletion (77 genes) and showed an insertion of 5654 bp, which contained different fragments encoding for Gyfsy-2 prophage proteins, UMUC protein and two fragments encoding for three Fels-2 prophage proteins (Figure A1). The deletion types *ΔfljAB*2-6, *ΔfljAB*8-9 and *ΔfljAB*12 varied in the length of their deletions, and they were all characterized by the insertion of one IS26 copy (Figure A2 and Figure A3). In the deletion *ΔfljAB*7, two IS26 copies were inserted (Figure A4). The deletion *ΔfljAB*10 had the largest insertion (7663 bp), which consisted of three insertion sequences (IS1, IS10 and IS26) and other additional genes, including tetracycline resistance genes (Figure A5). The main characteristic of *ΔfljAB*11 was the insertion of a truncated IS1 and a complete IS26 copy (Figure A2). Finally, in the deletion type *ΔfljAB*13, three variants of *fljB*-positive strains were included. These strains showed an insertion of one or two copies of IS26, generating a partial deletion, an interruption of the *hin* gene or were located after it (Figure A6). The deletion types belonging to *ΔfljAB*2-3, *ΔfljAB*6 and *ΔfljAB*8-13 had the IS26 copy, in the 3′-5′ direction, inserted in the same nucleotide of the intergenic region between the *hin* and *iroB* genes (the same ending point). However, the *ΔfljAB*4 and *ΔfljAB*5 deletions had the IS26 copy, in the 5′-3′ direction, inserted 222 bp downstream of the STM2757 gene (the same starting point).

## 4. Discussion

The emergence of the 4,[5],12:i:- monophasic variant of *Salmonella* Typhimurium demonstrates its evolutionary success. It has rapidly become one of the most prevalent serovars in humans in numerous countries worldwide [34]. The loss of the second-phase flagella has not prevented the emergence and worldwide spread of *S.* 4,[5],12:i:- monophasic variant strains. Flagella (H antigen) on the surface of *S.* Typhimurium had been characterized as a virulence factor that helps the bacteria move toward and adhere to host cells. However, Lockman and Curtiss [35] concluded that independent *Tn10* insertions that were mapped to different flagellar genes did not affect the virulence of *S.* Typhimurium for mice and suggested that motility might be irrelevant as a virulence factor for an invasive, facultative intracellular pathogen.

Classification of *Salmonella* by serotyping is generally performed by accredited National Reference Centers, as an essential epidemiological tool. In case of *S*. 4,[5],12:i:-, this procedure is crucial, since the non-expression or non-detection of the first and second flagellar antigens leads to the erroneous typing of *S*. Typhimurium as its monophasic variant. To solve this problem, the bacteria should be sequentially subcultivated for a new serotyping and, in case of negative results, an additional multiplex PCR should be completed [5]. Since this method is highly time-consuming and entails an unnecessary manipulation of the pathogen, multiplex PCR is routinely used. This PCR amplifies the *fljB-fljA* intergenic region of the flagellin gene cluster [5] but is unable to differentiate the monophasic *fljB*-positive variant from *S*. Typhimurium [36]. In this study, we found that WGS can prevent the amplification of the long-inserted fragments in the *fljAB* operon. Since most of the monophasic variant studies seldomly search for the deletion of the second flagellar phase, a complete strain characterization often requires applying multiple techniques. However, as we have verified through this work, NGS technologies allow a complete characterization of *Salmonella* strains within a few days. As an alternative to WGS, in 2018, a liquid bead array was proposed for the identification and characterization of *S*. 4,[5],12:i:- variants to achieve results in a rapid and simple data analysis [36]. In this work, we demonstrate that WGS can also be a rapid method that enhances traditional profiling efforts for the characterization of the monophasic variant, including the prediction of clinically relevant phenotypic traits such as antibiotic resistance genes, plasmids or virulence genes. On the other hand, the specific matrix of bead arrays only allows us to discriminate between *S.* Typhimurium and *S.* 4,[5],12:i:-, whilst we observed that WGS allows a detailed characterization of the second flagellar phase genetic deletions involved in this serovar.

To achieve full molecular description of strains, the development of efficient, standardized and molecular-guided laboratory surveillance is necessary and a high priority [37]. As presented in this work, WGS and freely available web services and bioinformatic tools can be extremely useful for public health laboratories and epidemiological surveillance. For instance, the bioinformatic tools used in this work allowed the achievement of the complete typing of *S*. 4,[5],12:i:-. Regarding the serotype, SeqSero2 has been considered more reliable for the serological prediction of the monophasic variant of *S.* Typhimurium, compared to other tools such as MOST and SISTR [38]. Even so, our results indicate that SeqSero2 does not correctly predict the serotype of *S*. 4,[5],12:i:- *fljB*-positive strains. In our study, *S*. 4,[5],12:i:- *fljB*-negative strains were classified 100% correctly by SeqSero2, but all *fljB*-positive monophasic strains were erroneously classified as *S*. Typhimurium. These results were verified by complete characterization of the *fljAB* operon based on genome assemblies. Similar results have been reported in other studies when assessing the serotyping of *S*. 4,[5],12:i:- strains using this program [39,40]. This limitation of SeqSero2 may be crucial for public health laboratories since *S*. Typhimurium and *S*. 4,[5],12:i:- are the most frequent human clinical infections after *S*. Enteritidis [2]. For this reason, as suggested by the comparative study done by Banerji et al. [39], we find it necessary to include additional factors to the *fljB* gene that determine the integrity of the second-phase flagellar antigen to detect *S.* 4,[5],12:i:- *fljB*-positive strains.

The in silico typing done in this work showed that, although WGS analysis seems expensive and complex, bioinformatic tools have transformed it into a cost-effective tool. Furthermore, large amounts of time and materials would be required if all typing had to be done through classic microbiology. To ensure the success of in silico typing, it is essential to generate high-quality contigs, which in turn requires evaluating sequence quality and the existence of possible technical errors by establishing quality control measures. Assessing genome assembly quality is significant in this process because poor-quality assemblies hamper downstream analyses, resulting in incorrect interpretations [37]. As such, it is critical to identify, evaluate and minimize technical errors occurring during sample isolation, DNA preparation sequencing and genome assembly.

Regardless of the epidemiological information, the study of the second flagellar phase deletions could provide further insight into their origin, the genetic events yielding them and the characterization of the inserted fragment. Through WGS carried out in this work, 13 different deletion types and subtypes of the second-phase flagellar genomic region were found in 32 *S*. 4,[5],12:i:- strains. Genetic diversity observed in the deletion types and in the in silico typing achieved by bioinformatic tools (ResFinder, SPIFinder, PlasmidFinder, PHASTER, in silico PCR and cgMLSTFinder) prove that the selection of strains analyzed in this work is a representation of non-clonal monophasic strains circulating in Spain.

The deletion Δ*fljAB*1, where 77 genes were absent compared to the genome of *S*. Typhimurium LT2 strain, showed an insertion of 5654 bp. The sequence of this insertion is very similar to the insertion described by Soyer et al. in the US strains [10], although some differences could be detected, namely, a 5654 bp fragment inserted instead of a 7 kb fragment, and the presence of the complete STM2704 gene, which is partially deleted on US strains. Of the total number of strains analyzed, four strains of clinical origin had this deletion; nevertheless, they varied in phenotypic characteristics. Strain 692 had the aminoglycoside resistance gene *aac (6′)-laa* that is a cryptic gene in *Salmonella* and IncFIB(S) and IncFII(S) plasmids. In strain 705, the cryptic gene *aac(6′)-laa* and the tetracycline resistance gene *tet(B)* were detected but no plasmids. Given the information provided by WGS, we considered that these strains could belong to the same lineage as the American strains described by Soyer et al. in the US. However, the other two strains with this same deletion (697 and 702) had these characteristics: *cmlA, aac(6′)–laa, aph(3″)-lb, aph(6)-ld, aadA1, aadA2, Sul3, tet(B)* and *dfrA12* resistance genes (CSSuTTm multi-resistance profile) and the presence of IncR plasmids. Interestingly, these two strains have the same characteristics as the Southern Europe clone described by Mourão et al. [14]. The results suggest that the strains 692 and 705 of the American clonal line are the ancestors of the Southern Europe clone in Spain. Moreover, the strains 697 and 702 with the American deletion, IncR plasmids and CSSuTTm multi-resistance profile are the representation of the Southern Europe clone in Spain, a result of the acquisition of IncR plasmids.

In 2016, Garcia et al. described an *S*. 4,[5],12:i:- clonal lineage widespread in Germany, Switzerland and Italy, carrying a ASSuT tetra-resistance induced by IncH1 plasmids, which replaced the second-phase flagellar genomic region [18]. We found eight strains *S*. 4,[5],12:i:- with an ASSuT tetra-resistance but none of these strains had the multidrug resistance plasmid described by Garcia et al. In six of these strains, only one or two copies of IS26 were detected in the second-phase flagellar genomic region (deletions Δ*fljAB*6, Δ*fljAB*7, Δ*fljAB*9, Δ*fljAB*12 and Δ*fljAB*13) and the remaining two *S.* 4,[5],12:i:- ASSuT strains were classified as Δ*fljAB*10 deletion type, showing a 7663 bp fragment between STM2761 and *iroB* genes. This fragment was composed of IS1, IS26 and a truncated IS10, *tetA* and *tetR* genes, which are implicated in tetracycline resistance, as well as of genes that codified a hypothetical protein or JemC, JemB and JemA products. Similar genetic composition has been described on an STM plasmid (pSRC27-H) and in the *S*. Typhimurium genome (T000240 strain). The putative roles of these insertion sequences would be the following: IS1 would drag the genes that appear on the T000240 strain; IS10, possibly located on the pSRC27-H plasmid, would be inserted by recognizing the *tetA*, *tetR* and *jemC* genes, and thus partially deleting the hypothetical protein of the T000240 strain; lastly, IS26 would interrupt IS10.

To date, two different models have been proposed to explain why the insertion sequence IS26 generates deletions in the second-phase flagellar region of *S*. 4,[5],12:i:- strains. On the one hand, it is suggested that most of the *fljB*-negative *S*. 4,[5],12:i:- strains observed globally could have emerged from a common ancestor containing an IS26 copy at that specific position [41]. On the other hand, it is theorized that several independent insertions may have occurred in different genetic events where an IS26 copy recognizes a hotspot [16,18]. In our study, *S*. 4,[5],12:i:- strains isolated in Spain containing at least one IS26 copy in the second-phase flagellar deletion have shown a genetic variability both in the region adjacent to the 3′-end of IS26 and in the genotyping based on WGS (presence of resistance genes, pathogenicity islands, plasmids, prophages and cgMLST). The great diversity found shows that *S*. 4,[5],12:i:- strains isolated in Spain and containing an IS26 copy do not belong to a single clone.

In this research, 85.71% of strains containing an IS26 in the second flagellar phase deletion had this insertion sequence in the 3′-5′ direction. In these deletions, the region adjacent to the 3′-end of IS26 (deletion starting point) varied between strains, while the region adjacent to the 5′-end (deletion ending point) was conserved. The IS26 insertion was found in the same position (i.e., 334 nucleotides upstream from the *iroB* gene) in *fljB*-negative strains isolated in the USA, South Korea and some European countries [18,36]. It is noteworthy that three strains of our work were *fljB*-positives (i.e., Δ*fljAB*13), even though they had IS26 inserted. The remaining 14.29% of strains had an IS26 in the 5′- 3′ direction and they all had the same deletion starting point. Interestingly, in a previous work with 60 strains of the *S*. 4,[5],12:i:- Spanish clone, 93.60% of the isolates shared the same starting point but different deletion ending points [9]. All these deletions had an IS26 inserted in the 5′-3′ direction in nucleotide no. 1444 of the gene STM2758 [9], very close to the starting point of the Δ*fljAB*4 and Δ*fljAB*5 deletions. WGS results show that the 5′-end of IS26 generates conserved deletion ends whilst creating significant variability in the genomic region adjacent to the 3′-end of the IS26 insertions. This finding suggests that the IS26 insertion sequence has a recognition end at 5′ that can be inserted in certain areas depending on its direction. Furthermore, we propose that *S*. 4,[5],12:i:- strains are evolving from different *S.* Typhimurium strains with no close phylogenetic relationship through different genetic events in which at least one IS26 was involved, promoting a pool of monophasic variants that share a similar deletion due to the IS26 5′-end recognition.

Several research groups further characterized *S.* 4,[5],12:i:- strains, reporting the existence of non-clonal *S.* 4,[5],12:i:- circulating in other European countries, such as Belgium, Italy, France and Poland [19,20,42]. However, other studies have observed *S.* 4,[5],12:i:- clonal lineages in the United Kingdom, Italy, Germany and Switzerland [7,16,18]. The detection of clonal and non-clonal strains may depend on the objectives of the research carried out in the above works. Although greatly expanded *S.* 4,[5],12:i:- clonal lines have been reported worldwide, it is possible that in the future, new *S.* 4,[5],12:i:- strains will be detected with different deletions of the second-phase flagellar genomic region. New *S.* 4,[5],12:i:- strains will be generated from *S*. Typhimurium strains in different genetic events, especially genomic rearrangements mediated by IS26. Pigs have been the main animal reservoir for *S.* 4,[5],12:i:- for years [34,43]. Therefore, it can be deduced that the genetic events causing the deletion of the second flagellar phase of the *Salmonella* strains analyzed in our study probably occurred within pigs. In fact, *S.* 4,[5],12:i:- was reported amongst the three most frequent serotypes in pigs in 2017, together with *S.* Typhimurium and *S*. Derby [34], in agreement with previous European guidelines [5]. Even so, in 2018, *S.* 4,[5],12:i:- strains causing human salmonellosis were associated mainly with pig (39.6%) and broiler (43.4%) sources [2]. This indicates that there has been a considerable expansion of *S.* 4,[5],12:i:- colonization of other animal niches. Based on these data, in our opinion, the emergence in the coming years of new *S.* 4,[5],12:i:- strains carrying deletions of the second flagellar phase should be expected from pig products and cannot be neglected from other animal sources.

## 5. Conclusions

In conclusion, our study demonstrates that the availability of sequencing technologies and the development of bioinformatic tools turn NGS into a realistic alternative to traditional methods for the characterization of *S*. 4,[5],12:i:- strains. In addition, these tools were essential to study the genetic bases of the monophasic phenotype and to identify *S*. 4,[5],12:i:- American clonal line strains in Spain that would give rise to the Southern Europe clone due to the acquisition of the IncR plasmid. Therefore, these tools were useful in determining the implication of the insertion sequence IS26 when generating new deletions and to establish the genetic link between *S*. 4,[5],12:i:- and *S*. Typhimurium strains. The results obtained in our study suggest that *Salmonella* monophasic variants are evolving from different *S*. Typhimurium strains through independent genetic events that may have taken place in swine. Within these genetic events, at least one IS26 was inserted whose 5′-end recognized a hotspot in the second-phase flagellar genomic region and generated conserved deletion ends. Finally, we consider that the genetic diversity observed in *S*. 4,[5],12:i:- strains analyzed in this study proves that non-clonal monophasic strains are circulating in Spain. Further studies are needed to analyze the recognition mechanism of the insertion sequence IS26 in the second-phase flagellar genomic region of *S*. 4,[5],12:i:-. This finding would help in the understanding of the mechanism by which new *S*. 4,[5],12:i:- strains are continuing to emerge.

## Figures and Tables

**Table 1:** Genotyping results from the open source bioinformatic tools on the 42 *Salmonella* isolates sequenced by whole genome sequencing (WGS).

Isolate Code	SeqSero2	INNUca MLST	cgMLSTFinder	ResFinder profile	SPIFinder	PlasmidFinder	PHASTER	*In Silico* IS26 PCR
692	4,[5],12:i:-	19	123420	S	SPI-1, SPI-3, SPI-5, SPI-13, C63PI	IncFIB(S), IncFII(S)	G2, EGF2, F2	−
693	4,[5],12:i:1,2	34	84985	ASSuT	SPI-1, SPI-2, SPI-3, SPI-5, SPI-13, SPI-14, C63PI	IncQ1	S3, G1, HP2, Ephi20	+
694	4,[5],12:i:1,2	34	78574	ASSuT	SPI-1, SPI-2, SPI-3, SPI-5, SPI-13, SPI-14, C63PI	IncQ1	S3, G1	+
695	4,[5],12:i:-	34	141108	ST	SPI-1, SPI-2, SPI-3, SPI-4, SPI-5, SPI-13, SPI-14, C63PI		S3, G1	+
696	4,[5],12:i:1,2	34	21377	SSuT	SPI-1, SPI-2, SPI-3, SPI-5, SPI-13, SPI-14, C63PI	IncQ1, IncFII	S3, G1, HP2, SfI	+
697	4,[5],12:i:-	19	85377	CSSuTTm	SPI-1, SPI-2, SPI-3, SPI-4, SPI-5, SPI-13, SPI-14, C63PI	IncR	G2, EGF2	+
698	4,[5],12:i:-	34	31310	AST	SPI-1, SPI-2, SPI-3, SPI-5, SPI-13, SPI-14, C63PI		S3, G1, HP2, SfI	+
699	4,[5],12:i:-	34	3719	ASSuT	SPI-1, SPI-2, SPI-3, SPI-5, SPI-13, SPI-14, C63PI	IncQ1, Col156	S3, G1, HP2, SfI	+
701	4,[5],12:i:-	34	141108	ST	SPI-1, SPI-2, SPI-3, SPI-5, SPI-13, C63PI		S3, G1	+
702	4,[5],12:i:-	19	85377	CSSuTTm	SPI-1, SPI-2, SPI-3, SPI-4, SPI-5, SPI-13, SPI-14, C63PI	IncR	G2, EGF2	+
703	4,[5],12:i:-	34	132646	ASSuT	SPI-1, SPI-2, SPI-3, SPI-5, SPI-13, SPI-14, C63PI		G2, HP2	+
704	4,[5],12:i:-	34	132646	ASSuT	SPI-1, SPI-2, SPI-3, SPI-4, SPI-5, SPI-13, SPI-14, C63PI	IncQ1	G1, HP2	+
705	4,[5],12:i:-	34	6912	ST	SPI-1, SPI-2, SPI-3, SPI-5, SPI-13, SPI-14, C63PI		G1	+
711	4,[5],12:i:-	19	156249	CSSuT	SPI-1, SPI-2, SPI-3, SPI-5, SPI-13, SPI-14, C63PI	IncI1, IncA/C2	G1, HP2	+
712	4,[5],12:i:-	19	156249	CSSuTTmNxCip	SPI-1, SPI-2, SPI-3, SPI-5, SPI-13, SPI-14, C63PI	Col(BS512), IncA/C2	G1, G2, HP2, EST104	+
713	4,[5],12:i:-	19	156249	ACSSuTTm	SPI-1, SPI-3, SPI-5, SPI-13, SPI-14, C63PI	Col(BS512), IncA/C2	G1, EST104	+
714	4,[5],12:i:-	19	156249	ACSSuTTm	SPI-1, SPI-2, SPI-3, SPI-5, SPI-14, C63PI	Col(BS512), IncA/C2	G1, G2, HP2, EST104	+
743	4,[5],12:i:-	34	132646	ASSu	SPI-1, SPI-3, SPI-4, SPI-5, SPI-13, SPI-14, C63PI	IncQ1	S3, G1, G2, HP2	+
744	4,[5],12:i:-	34	43443	ASSu	SPI-5, SPI-13, SPI-14, C63PI	IncQ1	S3, G1, G2, HP2	+
745	4,[5],12:i:-	34	165159	ASSuT	SPI-5, SPI-13, SPI-14, C63PI	IncQ1	S3, G1, G2, HP2, SfI	+
746	4,[5],12:i:-	34	132646	ASSu	SPI-1, SPI-2, SPI-3, SPI-4, SPI-5, SPI-13, SPI-14	IncQ1	G1	+
747	4,[5],12:i:-	34	100540	ASSuT	SPI-1, SPI-3, SPI-5, SPI-13, SPI-14, C63PI	IncQ1, Col440l	G1, Ep460	+
748	4,[5],12:i:-	34	26728	S	SPI-1, SPI-2, SPI-3, SPI-5, SPI-13, SPI-14, C63PI		G1, EfiAA91, SfII	+
749	4,[5],12:i:-	34	26728	ASSu	SPI-1, SPI-2, SPI-3, SPI-5, SPI-9, SPI-13, SPI-14, C63PI	IncQ1	S3, G1, EfiAA91, SfI	+
750	4,[5],12:i:-	34	26728	ASSu	SPI-1, SPI-2, SPI-3, SPI-5, SPI-13, SPI-14, C63PI	IncQ1	G1, EfiAA91, E186, SfI	+
751	4,[5],12:i:-	34	29699	CS	SPI-1, SPI-3, SPI-5, SPI-13, SPI-14, C63PI	IncI1	G1, E186, EfiAA91, SfII	+
752	4,[5],12:i:-	34	89891	CS	SPI-3, SPI-5, SPI-13, SPI-14, C63PI	IncI1	S3, G1, G2, E186, EfiAA91, ESfV	+
753	4,[5],12:i:-	34	144738	SSu	SPI-1, SPI-2, SPI-3, SPI-4, SPI-5, SPI-13, SPI-14, C63PI	IncQ1	S3, G1, G2, EST104, Epro147	+
754	4,[5],12:i:-	34	89891	S	SPI-1, SPI-3, SPI-5, SPI-13, SPI-14	IncI1	S3, G1, E186, EfiAA91, Ep460	+
755	4,[5],12:i:-	34	84985	ASSuT	SPI-1, SPI-2, SPI-3, SPI-5, SPI-13, SPI-14, C63PI	IncQ1	S3, G1, G2, HP2	+
757	4,[5],12:i:-	34	29699	CS	SPI-1, SPI-2, SPI-3, SPI-5, SPI-13, SPI-14, C63PI	IncI1	G1, G2	+
758	4,[5],12:i:-	34	144738	SSu	SPI-1, SPI-2, SPI-3, SPI-4, SPI-5, SPI-13, SPI-14, C63PI	IncQ1	G1, EST104, Epro147	+
739	4,[5],12:i:1,2	19	35732	S	SPI-1, SPI-2, SPI-3, SPI-5, SPI-13, SPI-14, C63PI	ColpVC, IncFIB(S), IncFII(S), IncX1	S3, G1, G2	−
756	4,[5],12:i:1,2	19	45281	ACSSuT	SPI-1, SPI-2, SPI-3, SPI-5, SPI-13, SPI-14, C63PI	IncFIB(S), IncFII(S)	EST104	−
759	4,[5],12:i:1,2	19	78568	ACSSuT	SPI-1, SPI-5, SPI-13, SPI-14, C63PI	IncX1, IncFII(S)	G1, G2, EST104	+
760	4,[5],12:i:1,2	19	20179	ACSSuTNxCip	SPI-1, SPI-2, SPI-3, SPI-5, SPI-13, SPI-14, C63PI	IncFII(S)	G1, G2, EST104, HP2	+
761	4,[5],12:i:1,2	19	35732	S	SPI-1, SPI-2, SPI-3, SPI-4, SPI-5, SPI-13, SPI-14, C63PI	ColpVC	S3, G1, G2	−
767	4,[5],12:i:1,2	19	45281	ACSSuT	SPI-1, SPI-2, SPI-3, SPI-4, SPI-5, SPI-13, SPI-14, C63PI	IncFIB(S), IncFII(S)	G1, G2, EST104	−
773	4,[5],12:i:1,2	19	45281	ACSSuT	SPI-1, SPI-2, SPI-3, SPI-4, SPI-5, SPI-13, SPI-14, C63PI	IncFIB(S), IncFII(S)	S3, G1, G2, EST104	−
775	4,[5],12:i:1,2	19	45281	ACSSuT	SPI-1, SPI-2, SPI-3, SPI-4, SPI-5, SPI-13, SPI-14, C63PI	IncFIB(S), IncFII(S)	S3, G1, G2, EST104	−
778	4,[5],12:i:1,2	19	35732	S	SPI-1, SPI-2, SPI-3, SPI-4, SPI-5, SPI-13, SPI-14, C63PI	ColpVC, IncFIB(S), IncFII(S), IncX1	G1, G2	−
779	4,[5],12:i:1,2	34	95263	ASSuTCol	SPI-1, SPI-3, SPI-5, SPI-13, SPI-14, C63PI	IncQ1, IncHI2, IncHI2A	S3, G1	+

N/A: The predicted antigenic profile does not exist in the White–Kauffmann–LE Minor Scheme; A: Amoxyciline (beta-lactamic); C: Chloramphenicol (phenicol); S: Streptomycin (aminoglycoside); Su: Sulphonamide; T: Tetracycline; Tm: Trimethoprim; Nx: Nalidixic acid (quinolone); Cip: Ciprofloxacin (fluoroquinolone); Col: Colistin (polymyxine); EGF2: Edward_GF2; E186: Entero_186; EST104: Entero_ST104; EfiAA91: Entero_fiAA91; Ep460: Entero_mEp460; ESfV: Entero_SfV; Ephi20: Entero_phi20; Epro147: Escher_pro147; F2: Fels_2; G1: Gifsy1; G2: Gifsy2; HP2: Haemoph_HP2; S3: Sal3; SfI: Shigel_SfI; SfII: Shigel_SfII; +: amplified; -: not amplified.

**Table 2:** Comparative results between SeqSero2 and classical serotyping, and between ResFinder genes and classical antimicrobial susceptibility tests.

Isolate Code	SeqSero2	Serotyping	ResFinder		Antibiogram Resistance Profile
			Antibiotic Resistance Genes and Mutations	Profile	
692	4,[5],12:i:-	4,[5],12:i:-	*aac(6′)-laa*	S	Susceptible
693	4,[5],12:i:1,2	4,[5],12:i:-	*blaTEM-1B; aac(6’)-laa; aph(3″)-lb; aph(6)-ld; Sul2; tet(B)*	ASSuT	AS^I^SuT
694	4,[5],12:i:1,2	4,[5],12:i:-	*blaTEM-1B; aac(6´)-laa; aph(3″)-lb; aph(6)-ld; Sul2; tet(B)*	ASSuT	ASSuT
695	4,[5],12:i:-	4,[5],12:i:-	*aac(6´)-laa; tet(B)*	ST	T
696	4,[5],12:i:1,2	4,[5],12:i:-	*aac(6´)-laa; aph(3″)-lb; Sul2; tet(B)*	SSuT	SSuT
697	4,[5],12:i:-	4,[5],12:i:-	*cmlA1; aac(6´)-laa; aph(3″)-lb; aph(6)-ld; aadA1; aadA2; Sul3; dfrA12; tet(B)*	CSSuTTm	T
698	4,[5],12:i:-	4,[5],12:i:-	*blaTEM-1B; aac(6´)-laa; tet(B)*	AST	AT
699	4,[5],12:i:-	4,[5],12:i:-	*blaTEM-1B; aac(6´)-laa; aph(3″)-lb; aph(6)-ld; Sul2; tet(B)*	ASSuT	ASSuT
701	4,[5],12:i:-	4,[5],12:i:-	*aac(6´)-laa; tet(B)*	ST	S^I^T
702	4,[5],12:i:-	4,[5],12:i:-	*cmlA1; aac(6´)-laa; aph(3″)-lb; aph(6)-ld; aadA1; aadA2; Sul3; dfrA12; tet(B)*	CSSuTTm	S^I^SuT
703	4,[5],12:i:-	4,[5],12:i:-	*blaTEM-1B; aac(6´)-laa; aph(3″)-lb; aph(6)-ld; Sul2; tet(B)*	ASSuT	ASSuT
704	4,[5],12:i:-	4,[5],12:i:-	*blaTEM-1B; aac(6´)-laa; aph(3″)-lb; aph(6)-ld; Sul2; tet(B)*	ASSuT	ASSuT
705	4,[5],12:i:-	4,[5],12:i:-	*aac(6´)-laa; tet(B)*	ST	ASSuT
711	4,[5],12:i:-	4,[5],12:i:-	*cmlA1; aac(6′)-Iaa; aadA8b; Sul1; Sul2; Sul3; tet(A)*	CSSuT	T
712	4,[5],12:i:-	4,[5],12:i:-	*cmlA1; aac(6′)-Iaa; Sul1; Sul2; Sul3; dfrA12; tet(A); gyrB p.E466D* mutation	CSSuTTmNxCip	SuT
713	4,[5],12:i:-	4,[5],12:i:-	*blaTEM-1B; cmlA1; aac(3)-IV; aac(6′)-Iaa; Sul1; Sul2; Sul3; dfrA12; tet(A)*	ACSSuTTm	ASSuT
714	4,[5],12:i:-	4,[5],12:i:-	*blaTEM-1B; cmlA1; aac(3)-IV; aac(6′)-Iaa; Sul1; Sul2; Sul3; dfrA12; tet(A)*	ACSSuTTm	ASSuT
743	4,[5],12:i:-	4,[5],12:i:-	*blaTEM-1B; aac(6′)-laa; aph(3″)-lb; aph(6)-ld; Sul2*	ASSu	ASSu
744	4,[5],12:i:-	4,[5],12:i:-	*blaTEM-1B; aac(6′)-laa; aph(3″)-lb; aph(6)-ld; Sul2*	ASSu	ASSu
745	4,[5],12:i:-	4,[5],12:i:-	*blaTEM-1B; aac(6′)-laa; aph(3″)-lb; aph(6)-ld; Sul2; tet(B)*	ASSuT	ASSuT
746	4,[5],12:i:-	4,[5],12:i:-	*blaTEM-1B; aac(6′)-laa; aph(3″)-lb; aph(6)-ld; Sul2*	ASSu	ASSu
747	4,[5],12:i:-	4,[5],12:i:-	*blaTEM-1B; aac(6′)-laa; aph(3″)-lb; aph(6)-ld; Sul2; tet(B)*	ASSuT	ASSuT
748	4,[5],12:i:-	4,[5],12:i:-	*aac(6′)-laa*	S	Susceptible
749	4,[5],12:i:-	4,[5],12:i:-	*blaTEM-1B; aac(6′)-laa; aph(3″)-lb; aph(6)-ld; Sul2*	ASSu	ASSu
750	4,[5],12:i:-	4,[5],12:i:-	*blaTEM-1B; aac(6′)-laa; aph(3″)-lb; aph(6)-ld; Sul2*	ASSu	A
751	4,[5],12:i:-	4,[5],12:i:-	*floR; aac(3)-IV; aac(6′)-laa; aph(3″)-lb; aph(4)-la; aph(6)-ld*	CS	ACS
752	4,[5],12:i:-	4,[5],12:i:-	*floR; aac(3)-IV; aac(6′)-laa; aph(3″)-lb; aph(4)-la; aph(6)-ld*	CS	ACSSu
753	4,[5],12:i:-	4,[5],12:i:-	*aac(6′)-laa; aph(3″)-lb; aph(6)-ld; Sul2*	SSu	ACS^I^SuT
754	4,[5],12:i:-	4,[5],12:i:-	*aac(3)-IV; aac(6′)-laa; aph(3″)-lb; aph(4)-la; aph(6)-ld*	S	AS
755	4,[5],12:i:-	4,[5],12:i:-	*blaTEM-1B; aac(6′)-laa; aph(3″)-lb; aph(6)-ld; Sul2; tet(B)*	ASSuT	ASSuT
757	4,[5],12:i:-	4,[5],12:i:-	*floR; aac(3)-IV; aac(6′)-laa; aph(3″)-lb; aph(4)-la; aph(6)-ld*	CS	CS
758	4,[5],12:i:-	4,[5],12:i:-	*aac(6′)-laa; aph(3″)-lb; aph(6)-ld; Sul2*	SSu	SSu
739	4,[5],12:i:1,2	4,[5],12:i:1,2	*aac(6′)-laa*	S	Susceptible
756	4,[5],12:i:1,2	4,[5],12:i:1,2	*blaCARB-2; floR; aac(6′)-laa; aadA2; Sul1; tet(G)*	ACSSuT	ACSSuT
759	4,[5],12:i:1,2	4,[5],12:i:1,2	*blaOXA-1; catA1; aac(6′)-laa; aadA1; Sul1; tet(B)*	ACSSuT	ACSSuT
760	4,[5],12:i:1,2	4,[5],12:i:1,2	*blaOXA-1; catA1; aac(6′)-laa; aadA1; Sul1; tet(B); gyrA p. D87N* mutation	ACSSuTNxCip	ACSSuTNx
761	4,[5],12:i:1,2	4,[5],12:i:1,2	*aac(6′)-laa*	S	Susceptible
767	4,[5],12:i:1,2	4,[5],12:i:1,2	*blaCARB-2; floR; aac(6′)-laa; aadA2; Sul1; tet(G)*	ACSSuT	ACSSuT
773	4,[5],12:i:1,2	4,[5],12:i:1,2	*blaCARB-2; floR; aac(6′)-laa; aadA2; Sul1; tet(G)*	ACSSuT	ACSSuT
775	4,[5],12:i:1,2	4,[5],12:i:1,2	*blaCARB-2; floR; aac(6′)-laa; aadA2; Sul1; tet(G)*	ACSSuT	ACSSuT
778	4,[5],12:i:1,2	4,[5],12:i:1,2	*aac(6′)-laa*	S	Susceptible
779	4,[5],12:i:1,2	4,[5],12:i:1,2	*blaTEM-1B; blaCTX-M-9; aac(6′)-laa; aadA2; aph(3″)-lb; aph(6)-ld; Sul1; Sul2; dfrA16; tet(A); mcr-9*	ASSuTCol	ASSuTNxCfx

N/A: The predicted antigenic profile does not exist in the White–Kauffmann–LE Minor Scheme; A: Amoxyciline (beta-lactamic); C: Chloramphenicol (phenicol); S: Streptomycin (aminoglycoside); Su: Sulphonamide; T: Tetracycline; Tm: Trimethoprim; Nx: Nalidixic acid (quinolone); Cip: Ciprofloxacin (fluoroquinolone); Col: Colistin (polymyxine); Cfx: Cefotaxime (third generation cephalosporin); S^I^: Intermediate susceptibility to streptomycin.

**Table 3:** Description of the *fljAB* operon deletion types of the 32 *S*. 4,[5],12:i:-analyzed. In *fljB*-negative types (Δ*fljAB*1–12) the starting and ending points of the deletions have been specified.

Deletion Type	Subtype	No. of Strains	Starting Point	Ending Point	Inserted Fragment ^†^
Δ*fljAB*1		4	98 bp of STM2693	10 bp downstream of STM2771 (*fljB*)	5654 bp (see Figure A1)
Δ*fljAB*2	Δ*fljAB*2-A	1	1201 bp of STM2746	334 bp upstream from STM2773 (*iroB*)	820 bp (one IS26)
	Δ*fljAB*2-B	1	1263 bp of STM2746		
Δ*fljAB*3	Δ*fljAB*3-A	1	177 of STM2753	334 bp upstream from STM2773 (*iroB*)	820 bp (one IS26)
	Δ*fljAB*3-B	1	207 bp of STM2753		
	Δ*fljAB*3-C	1	353 bp of STM2753		
Δ*fljAB*4		3	222 bp downstream of STM2757	571 bp of STM2773 (*iroB*)	820 bp (one IS26)
Δ*fljAB*5		1	222 bp downstream of STM2757	848 bp of the STM2784	820 bp (one IS26)
Δ*fljAB*6	Δ*fljAB*6-A	1	1079 bp of STM2759	334 bp upstream from STM2773 (*iroB*)	820 bp (one IS26)
	Δ*fljAB*6-B	2	142 bp downstream of the STM2759		
Δ*fljAB*7		1	998 bp downstream of STM2759	475 bp of STM2774 (*iroC*)	1640 bp (two IS26)
Δ*fljAB*8		7	88 bp of STM2760	334 bp upstream from STM2773 (*iroB*)	820 bp (one IS26)
Δ*fljAB*9		1	175 bp of the STM2761	334 bp upstream from STM2773 (*iroB*)	820 bp (one IS26)
Δ*fljAB*10		2	1125 bp of the STM2761	334 bp upstream from STM2773 (*iroB*)	7648 bp (see Figure A5)
Δ*fljAB*11		1	118 bp downstream of STM2767	334 bp upstream from STM2773 (*iroB*)	1455 bp (see Figure A2)
Δ*fljAB*12		1	155 bp of the *fljA* gene	334 bp upstream from STM2773 (*iroB*)	820 bp (one IS26)
Δ*fljAB*13	Δ*fljAB*13-A	1	*fljB*-positive		1640 bp (two IS26)
	Δ*fljAB*13-B	1			
	Δ*fljAB*13-C	1			820 bp (one IS26)

† Detailed information is in appendix Figure A1, Figure A2, Figure A3, Figure A4, Figure A5 and Figure A6.

## Appendix A

**Table A1:** *Salmonella* isolates selected to this work.

Isolate Code	Year	Source	Origin	Serovar	Resistance Profile	Phage Type
692	2008	Human feces	NCM	4,[5],12:i:-	Susceptible	104b
693	2008	Human feces	NCM	4,[5],12:i:-	AS^I^SuT	193
694	2008	Human feces	NCM	4,[5],12:i:-	ASSuT	195
695	2008	Human feces	NCM	4,[5],12:i:-	T	138
696	2008	Human feces	NCM	4,[5],12:i:-	SSuT	NRP
697	2008	Human feces	NCM	4,[5],12:i:-	T	NRP
698	2008	Human feces	NCM	4,[5],12:i:-	AT	104b
699	2008	Human feces	NCM	4,[5],12:i:-	ASSuT	193
701	2008	Human feces	NCM	4,[5],12:i:-	S^I^T	138
702	2008	Human feces	NCM	4,[5],12:i:-	S^I^SuT	NRP
703	2008	Human feces	NCM	4,[5],12:i:-	ASSuT	7
704	2008	Human feces	NCM	4,[5],12:i:-	ASSuT	NRP
705	2008	Human feces	NCM	4,[5],12:i:-	ASSuT	NRP
711	1999	Chicken sausage	PHL	4,[5],12:i:-	T	ND
712	2000	Chicken sausage	PHL	4,[5],12:i:-	SuT	ND
713	2000	Chicken sausage	PHL	4,[5],12:i:-	ASSuT	ND
714	2000	Chicken sausage	PHL	4,[5],12:i:-	ASSuT	ND
743	2012	Swine IC	IdAB	4,[5],12:i:-	ASSu	U311
744	2012	Swine MLN	IdAB	4,[5],12:i:-	ASSu	ND
745	2012	Swine MLN	IdAB	4,[5],12:i:-	ASSuT	ND
746	2012	Swine IC	IdAB	4,[5],12:i:-	ASSu	ND
747	2012	Swine IC	IdAB	4,[5],12:i:-	ASSuT	ND
748	2015	Swine IC	IdAB	4,[5],12:i:-	Susceptible	ND
749	2015	Swine IC	IdAB	4,[5],12:i:-	ASSu	ND
750	2015	Swine IC	IdAB	4,[5],12:i:-	A	ND
751	2015	Swine IC	IdAB	4,[5],12:i:-	ACS	ND
752	2015	Swine IC	IdAB	4,[5],12:i:-	ACSSu	ND
753	2015	Swine IC	IdAB	4,[5],12:i:-	ACS^I^SuT	ND
754	2015	Swine IC	IdAB	4,[5],12:i:-	AS	ND
755	2015	Swine IC	IdAB	4,[5],12:i:-	ASSuT	ND
757	2015	Swine IC	IdAB	4,[5],12:i:-	CS	ND
758	2015	Swine IC	IdAB	4,[5],12:i:-	SSu	ND
739	2012	Swine MLN	IdAB	4,[5],12:i:1,2	Susceptible	NRP
756	2012	Swine MLN	IdAB	4,[5],12:i:1,2	ACSSuT	ND
759	2012	Swine IC	IdAB	4,[5],12:i:1,2	ACSSuT	ND
760	2012	Swine MLN	IdAB	4,[5],12:i:1,2	ACSSuTNx	104b
761	2012	Swine MLN	IdAB	4,[5],12:i:1,2	Susceptible	NRP
767	2012	Swine MLN	IdAB	4,[5],12:i:1,2	ACSSuT	U302
773	2012	Swine MLN	IdAB	4,[5],12:i:1,2	ACSSuT	104b
775	2012	Swine MLN	IdAB	4,[5],12:i:1,2	ACSSuT	104b
778	2012	Swine MLN	IdAB	4,[5],12:i:1,2	Susceptible	ND
779	2012	Swine MLN	IdAB	4,[5],12:i:1,2	ASSuTNxCfx	ND

NCM: Spanish National Centre for Microbiology (Majadahonda, Spain); PHL: Public Health Laboratory (Zamudio, Spain); IdAB: Institute of Agrobiotechnology (Navarra, Spain); IC: Intestinal content; MLN: Mesenteric lymph nodes*;* A: Amoxyciline (beta-lactamic); C: Chloramphenicol (phenicol); S: Streptomycin (aminoglycoside); Su: Sulphonamide; T: Tetracycline; Nx: Nalidixic acid (quinolone); Cfx: Cefotaxime (third generation cephalosporin); S^I^: Intermediate susceptibility to streptomycin; NRP: Non-recognizable pattern; ND: Not determined.
